# Beyond the Surface: A Multidetector Computer Tomography Scan Investigation into Age and Gender Differences.

**DOI:** 10.12688/f1000research.156445.1

**Published:** 2025-01-15

**Authors:** Anu Vinod Ranade, Rajalakshmi Rai, Biswabina Ray, Soumya Vinod

**Affiliations:** 1 Basic Medical Sciences, College of Medicine, University of Sharjah, Sharjah, United Arab Emirates; 2 Cardiovascular Research Group, Sharjah Institute for Medical Research, University of Sharjah, Sharjah, United Arab Emirates; 3 Department of Anatomy, Kasturba Medical College Mangalore, Manipal Academy of Higher Education, Mangaluru, Karnataka, India; 4 Department of Anatomy, AIIMS, Kalyani, West Bengal, 741245, India; 5 Department of Anatomy, College of Medicine and Health Sciences, United Arab Emirates University, Al Ain, Abu Dhabi, United Arab Emirates

**Keywords:** air sinuses, forensic anthropology, morphometry, computed tomography, imaging.

## Abstract

**Background:**

The identification of an individual after mass calamities poses challenges to experts when bones are fragmented. Dense bones, such as the maxilla, surpass this challenge and remain intact with sinuses even after incineration, thereby making the sinuses an ideal and reliable forensic science tool. No-ninvasive imaging techniques, such as Computed Tomography (CT), can be used to evaluate such cases and help detect fractures and further locate foreign bodies. This study aimed to estimate the dimensions and volumes of the frontal (FS), maxillary (MS), and sphenoidal air sinuses (SS) on CT scans and investigate age- and sex-related differences.

**Methods:**

CT scans of the paranasal sinuses were acquired from 158 patients ranging in age from 19 to 73 years, and written consent was obtained from all participants. This study was approved by the Institutional Ethical Committee (ethical clearance number IEC 064/2010). All parameters were statistically analyzed using SPSS 20 version and the significance level was set at p<0.05.

**Results:**

The bilateral anteroposterior length and height of the FS were significantly larger in men than females (p<0.05). The overall dimensions of the MS and SS were substantially greater in males than females (P <0.05). Likewise, the overall volumes of the fFS, MS, and SS were significantly greater in males than females (p<0.05).

However, no significant age-related correlation was observed in the dimensions and volumes of the sinuses.

**Conclusion:**

The results of this study showed that imaging could be a reliable instrument for personal identification in forensic anthropology. Countries that do not allow autopsies may implement this method to clarify the cause of death.

## Introduction

“Nothing else will matter until she has a name,” a poignant observation by the renowned anthropologist Kathy Reich underscores the critical importance of personal identification in forensic investigations. ^1^ Establishing a victim’s identity, particularly following a mass disaster, presents a formidable challenge for forensic experts because of the overwhelming number of casualties and limitations of available resources. ^2^ A general guideline emphasizes the urgency of prompt victim identification because delays can significantly compromise the accuracy of this process. ^3^ Moreover, identifying the deceased holds profound significance for grieving family members, friends, and relatives. ^3^

To initiate the identification process, the first step involves assessing the body’s sexual dimorphism and age. ^4, 5^ While traditional methods, such as fingerprinting and DNA analysis, offer accurate results, they can be time-consuming and require specialized laboratories and equipment. These techniques may also be impractical when the body undergoes significant decomposition. ^6^ Forensic experts frequently utilize intact skeletal remains from the skull, pelvis, and long bones to determine the age and sex of victims. ^7^ However, in mass disasters such as tsunamis, earthquakes, explosions, and aircraft crashes, forensic experts may encounter fragmented skeletal remains. ^7^ The distinctive characteristics of certain bones, including the maxillary and zygomatic bones, can mitigate these challenges. Even in incineration cases, the sinuses of these bones may remain intact, rendering their dimensions a valuable and reliable tool for forensic identification. ^8^

Furthermore, maxillary sinus (MS) pneumatization differs between people, and its volume is affected by age. ^9^ Frontal sinuses (FS) are unique even amidst monozygotic twins, are unchangeable and persistent, and have sturdy walls that remain undamaged in human corpses. ^6, 10^ The sphenoid sinus (SS) pneumatization pattern varies in proportion and direction, and based on these anatomical features, it is used by clinical researchers in forensic investigations. ^11^ Additionally, detailed anatomical knowledge of the normal sinuses and their variants plays a vital role in avoiding complications during functional endoscopic sinus surgeries. ^10^ The utilization of cross-sectional imaging modalities, such as Computed Tomography (CT), Magnetic Resonance Imaging (MRI), CT angiography, and imaging-guided biopsy, has resulted in significant advancements in the medico-legal field, particularly with regard to identifying human remains. ^12– 14^ CT imaging allows for precise positioning and measurement of anthropometric points within the skull as well as the calculation of volumes and areas. ^14^ These images may be compared to antemortem records, such as medical or dental records, to establish positive identification. ^15^ Moreover, these noninvasive techniques enable the re-evaluation of cases and can assist in detecting air embolisms, fractures, and locating projectiles and foreign bodies. ^12, 16– 18^

Paranasal sinus analysis can also be used in conjunction with other methods of identification, such as DNA analysis or dental records, to strengthen identification. ^19^ It is also a non-invasive method of identification that does not require the removal of tissue or disturbance of the body. In countries where autopsies are not permitted, this method can be implemented to document potential injuries and elucidate the manner and cause of death. ^20^ As data related to skeletal measurements of air sinuses in deceased individuals are limited, recent studies have been conducted within the living population to record discriminant age- and sex-based data. ^21^

Given the diverse importance of the morphometry of the air sinuses, this study aimed to measure the dimensions and volumes of the FS, MS, and SS air sinuses on CT scans, as well as to investigate age- and sex-dependent differences.

## Methods

Ethical approval was obtained from the Manipal Academy of Higher Education through its’ Institutional Ethics Committee (Ethical clearance/ref no: 064). Prior to the study, all participants were requested to provide written consent for the use of their anonymized data in future research and publication.

This study utilized a retrospective cross-sectional design based on the STROBE guidelines {Cuschieri, 2019 #1}. Cranial CT images were obtained from158 patients of varying ages (19-73years), consisting of 79 males and 79 females, from whom prior consent was obtained. The sample size was determined using the calculation reported by Buyuk et al. ^22^

For morphometric measurements, patients with normal bony margins and paranasal sinuses without any signs of inflammation were included for volume measurements were included in the study. Patients with damaged bony margins and those with sinonasal pathologies, such as inflammatory changes or space-occupying lesions, were excluded.

A 64-row detector Philips Brilliance CT scanner was used to perform CT scans of the sinonasal region in a tertiary hospital. The helical CT study utilized a high-resolution bone algorithm with the following parameters: Pitch, 64×0.625:0.64, rotation: 0.5/sec, Fov: 180 mm, filter: bone (D), enhancement: nil, window: C200 W2000, matrix: 512, slice thickness: 0.9 mm, increment: 0.45 mm, KV: 120, mAS\slice: 100. The data obtained from the scan were isotropic, enabling further post-processing morphometry investigation. Sinus morphometric data were obtained by reforming the scans in the axial, sagittal, and coronal planes using isotropic volumetric data. Axial reformation was performed with the plane of reformation parallel to the hard palate, coronal reformation plane perpendicular to the vomer, and sagittal reformation plane parallel to the vomer.

**Figure 1.**
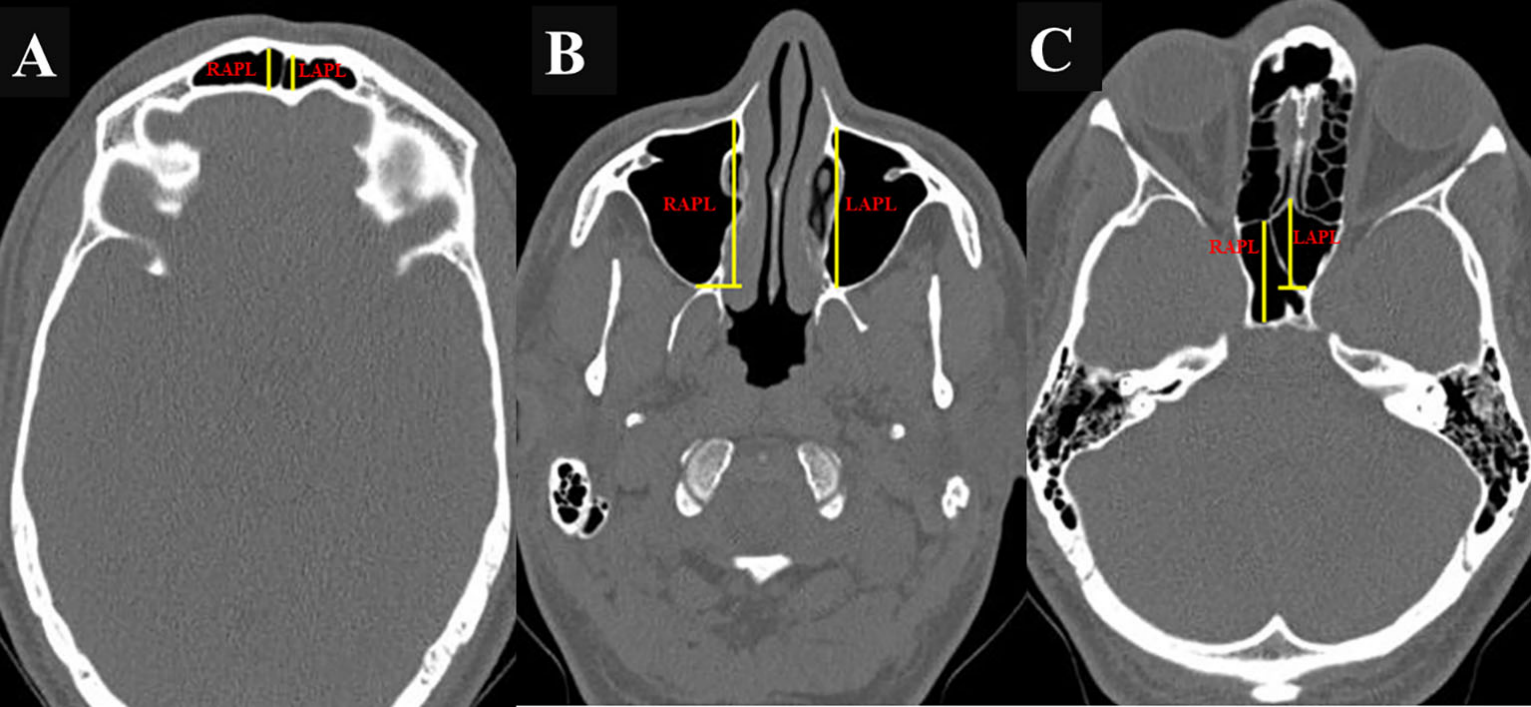
(A,B,C): Axial CT image showing the AP measurement of FS, MS and SS respectively. (RAPL- right anteroposterior length; LAPL- left anteroposterior length).

**Figure 2.**
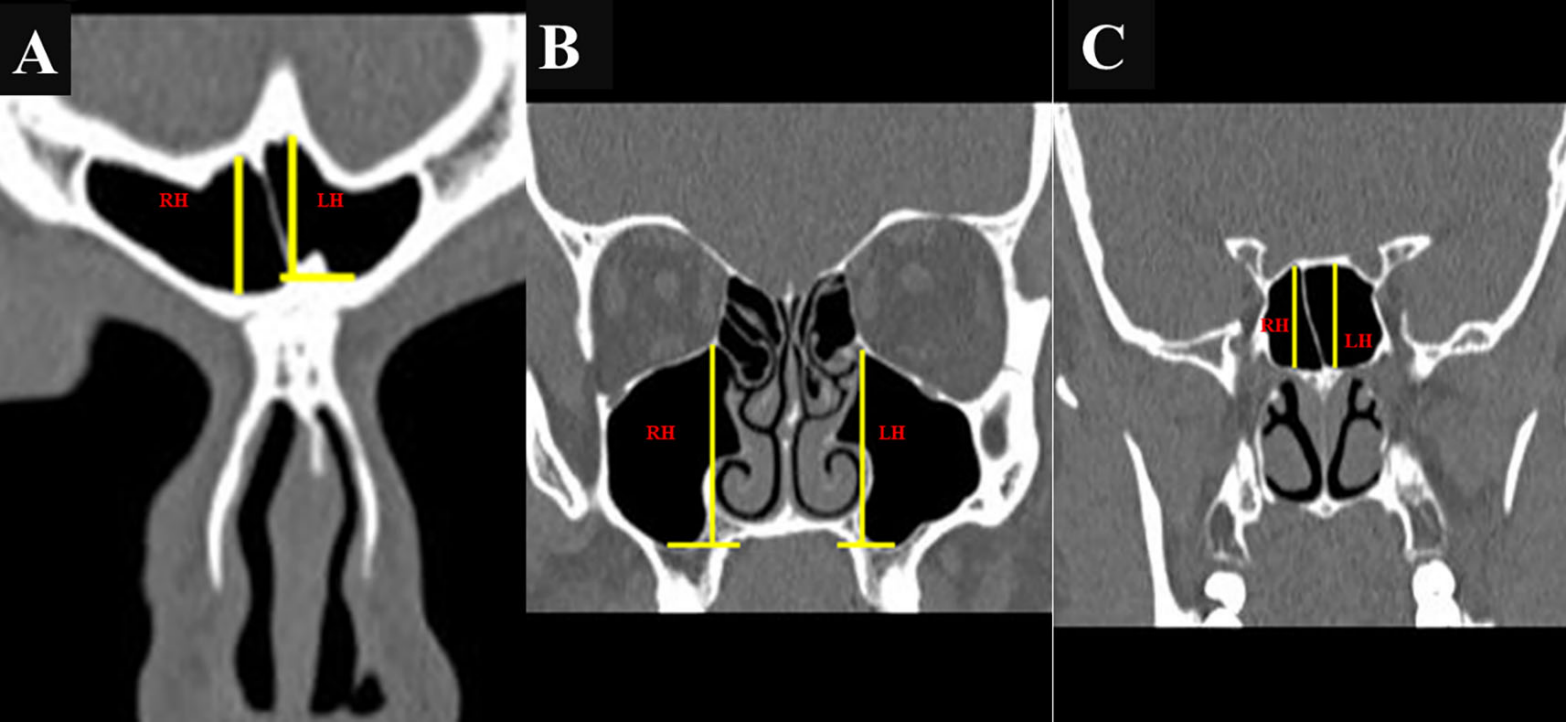
(A, B, C): Coronal CT images showing the height of FS, MS, and SS, respectively. (RH, right height; LH, left height).

**Figure 3.**
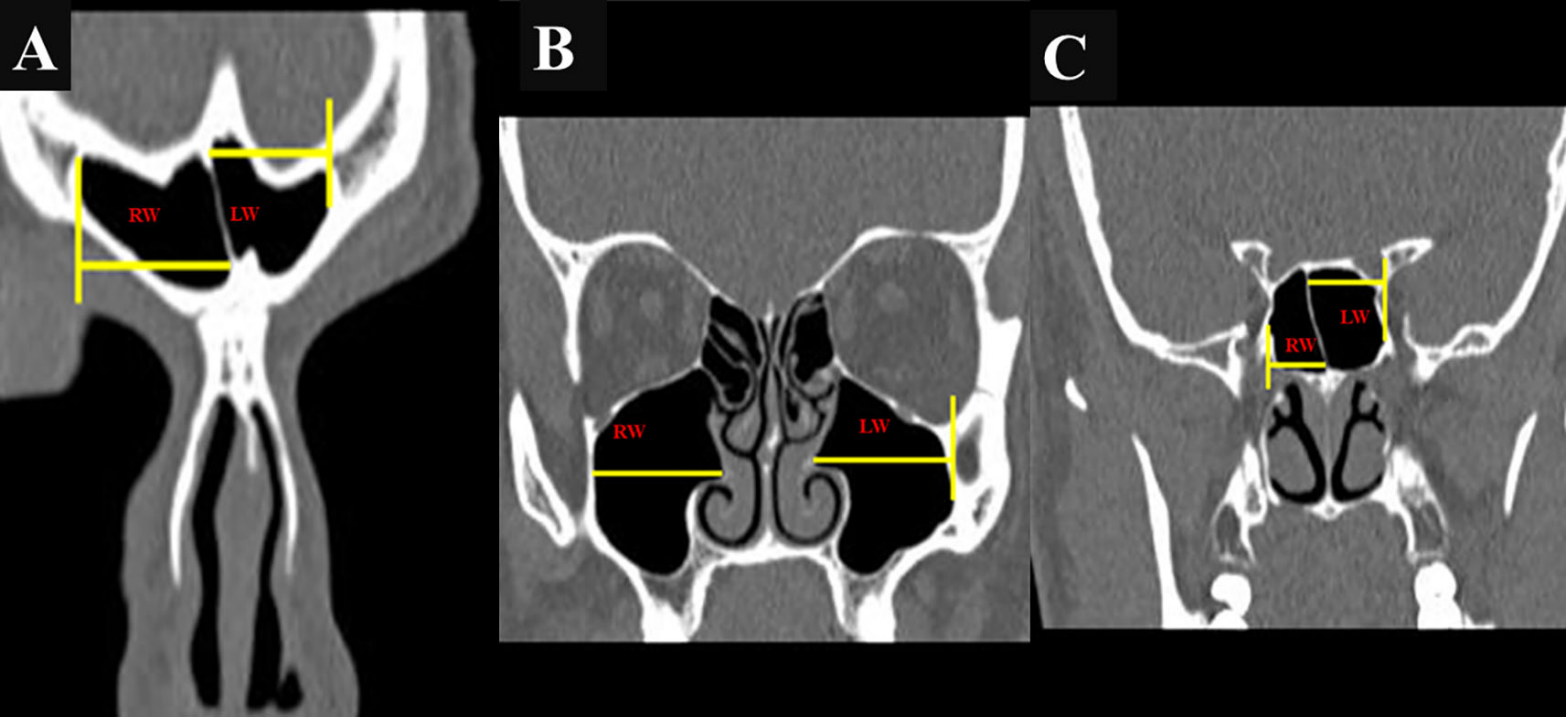
(A, B, C): Coronal CT image showing width measurements of FS, MS, and SS, respectively. (RW: right width; LW: left width).

The maximum anteroposterior (AP) length, maximum vertical height, and maximum width of the FS, MS, and SS were assessed using “Dicom software,” and the values were expressed in millimeters. Measurements of the FS, MS, and SS were obtained using DICOM software and expressed in millimeters. The maximum anteroposterior (AP) length was measured in the axial plane ( Figure 1A, 1B, and 1C), while the maximum vertical height ( Figure 2A, 2B, and 2C) and width ( Figure 3A, 3B, and 3C) were assessed in the coronal plane.

In the case of the FS, the AP length was measured from the most anterior to the most posterior point on the sinus in the axial plane ( Figure 1A), and the height was calculated from the lowest to the highest point on the coronal plane sinus ( Figure 2A). Width was measured as the longest distance between the medial and lateral points in the coronal plane ( Figure 3A).

For the MS, AP length was measured from the anterior-most point to the posterior-most point along the medial wall of the sinus in the axial plane ( Figure 1B). The height was measured from the bottom-most point on the sinus floor to the uppermost end of the roof of the sinus in the coronal plane (2 B), while the width was taken as the longest distance between the medial and lateral walls of the coronal plane’s sinus (3 B). For the SS, the septum was considered as the border between the two sinuses. AP length, height, and width were measured from the anterior-most point to the posterior-most point in the axial plane ( Figure.1C), the lowest to the highest point in the coronal plane ( Figure 2C), and the medial-most point in the coronal plane ( Figure 3C).

**Figure 4.**
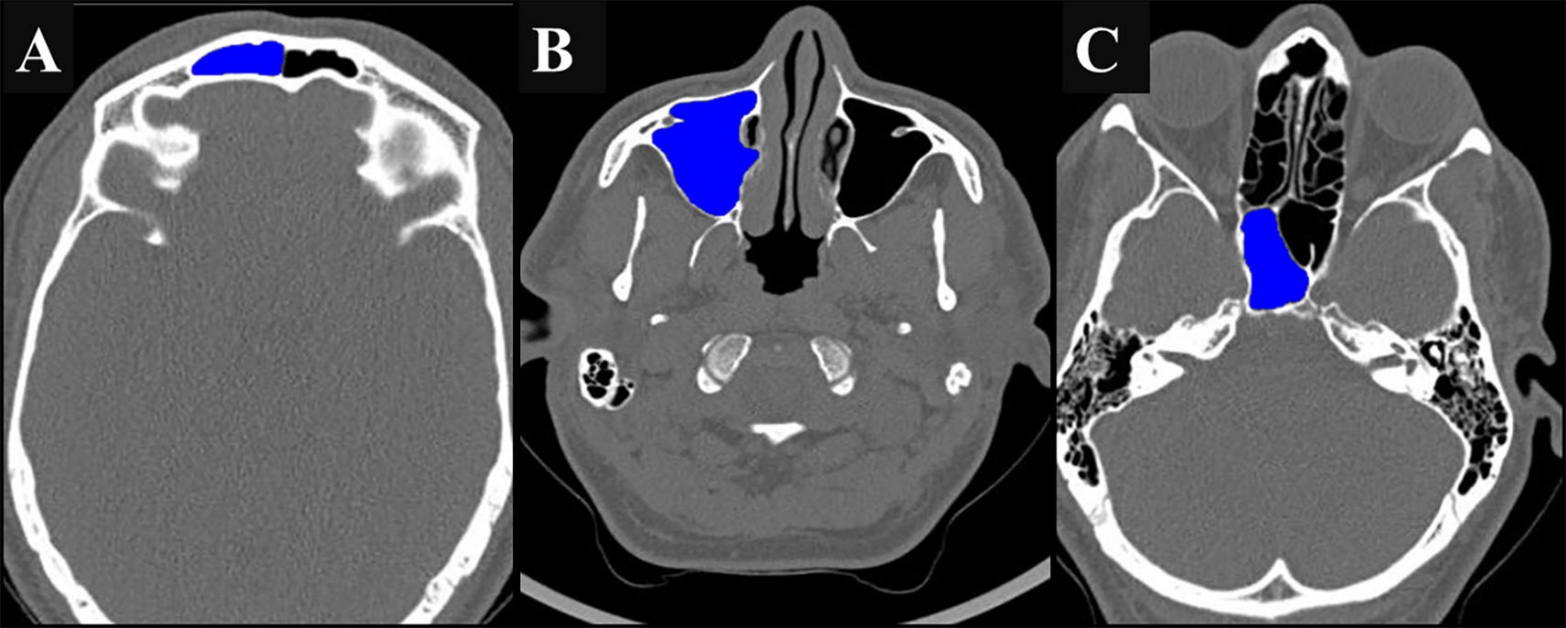
(A, B, C): Axial CT scan depicting volume measurement of the right frontal sinus (FS), maxillary sinus (MS), and sphenoid sinus (SS) using contrast dye injected into the sinus cavities.

The volumes of all three sinuses were calculated using the volume tracing tool software. A dye was injected into the sinus cavity for segmentation, and three-dimensional reconstruction of the filled sinus cavity was performed to calculate the volume of the sinus, which was expressed in cubic centimeters ( Figure 4A, 4B, and 4C).

### Statistical analysis

Pearson’s correlation test was applied to establish the correlation of the dimensions and volumes of the frontal, maxillary, and sphenoid sinuses with age. Independent samples t-test was used to compare sex, and statistical significance was set at p<0.05. The analysis was conducted using IBM SPSS 20.0 (IBM Inc., Chicago).

## Results

**Figure 5.**
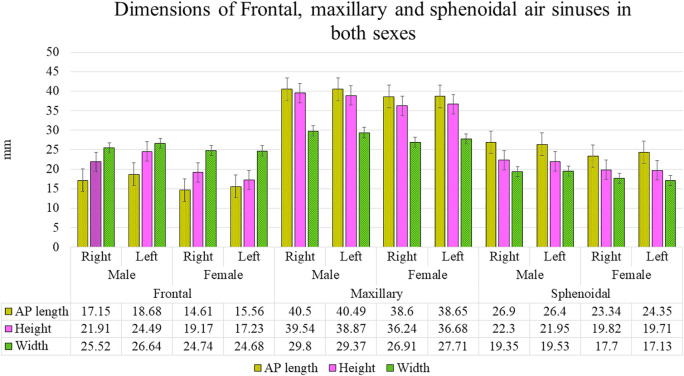
Volumes of frontal, maxillary and sphenoidal sinuses in both sexes.

**Figure 6.**
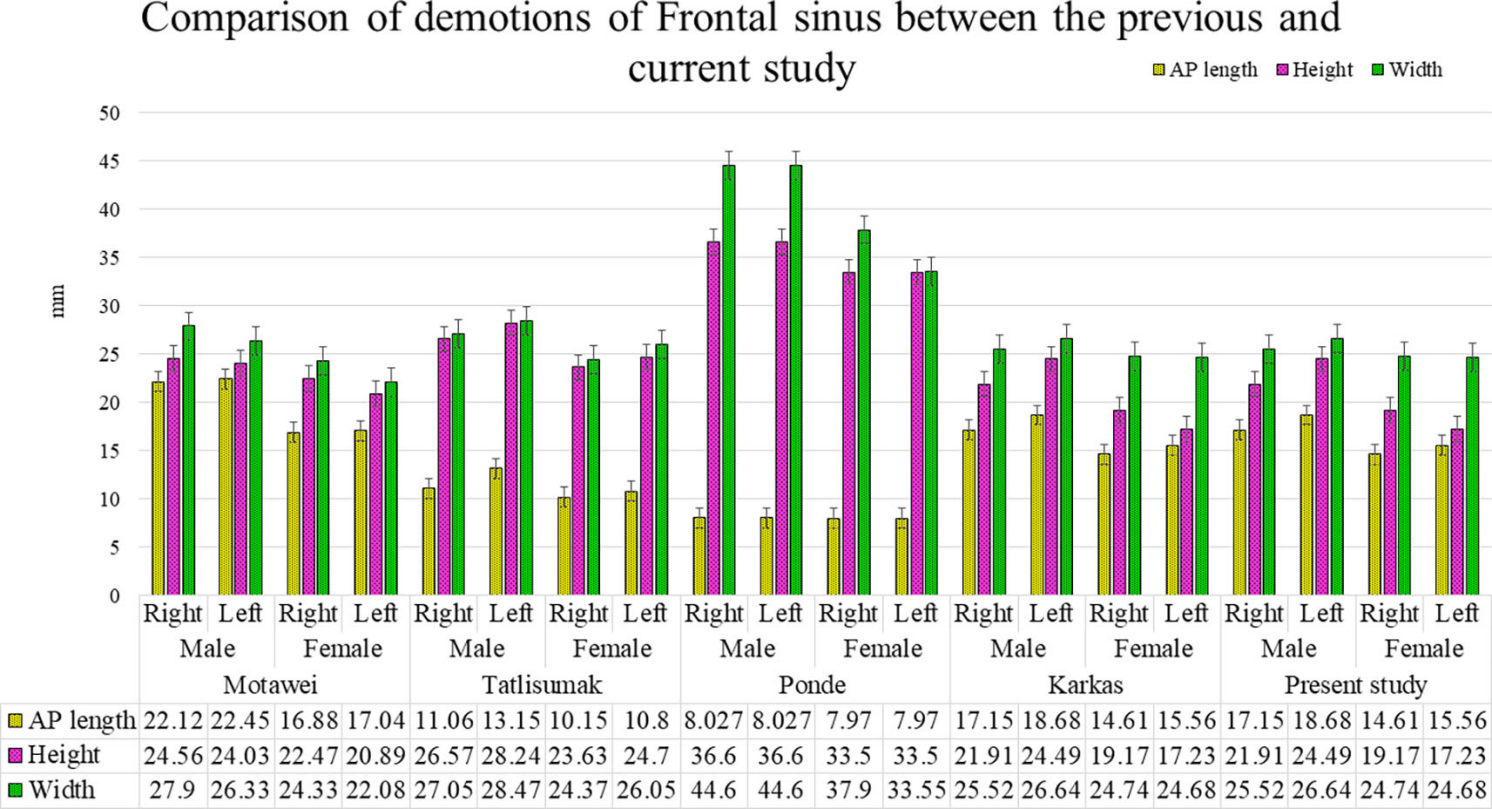
Dimensions of frontal, maxillary and sphenoidal sinuses in both sexes.

The descriptive statistical mean, standard deviation, and ‘P’ value using Student’s t-test for independent samples are tabulated in Figure 5, representing volume, and Figure 6, representing dimensions.

The AP length and height of the FS on both the right and left sides were greater among males (17.1±6.9; 21.9±7.9 and 18.6±6.4; 24.4±9.2 mm) than in females (14.6±5.6; 19.1±13.7; and 15.5±6.4; 17.2±6.9 mm).

The mean AP length of the FS on both sides was significantly greater in males than in females (p<0.05).

The mean heights of the FS on the right and left sides were larger in males than females, and the difference was significant on the left side (p<0.001). In addition, the mean width of the FS bilaterally was greater in males compared than in females, although this difference was not statistically significant.

We also found that the AP length, height, and width of the MS on the right and left sides were significantly greater in males (40.0 ±3.1; 39.5±5.1; 29.8±5.1 mm and 40.4±4.0; 38.8±5.8; 29.3±5.4 mm) when compared to the right and left sides in females (38.6±3.1; 36.2±6.0; 26.9±4.5 and 38.6±3.3; 36.6±5.14; 27.7±4.6 mm) (p<0.001). Similarly, the AP length, height, and width of the SS on both sides in males (Right 26.9±6.9; 22.3±4.8; 19.3±6.4 and Left 26.4±7.6; 21.9±5.2; 19.5±6.3 mm, respectively) were significantly greater than those in females (right 23.3±6.8, 19.8±4.3, and 17.7±5.2, respectively; and left 24.3±6.7, 19.7±4.2, and 17.1±4.6 mm, respectively).

We further measured the bilateral volume of FS (41 males and females, respectively), MS (40 males, 43 females), and SS (42 males, 61 females) in a subset of individuals and found that the volumes of all three paranasal sinuses were significantly greater in males than in females (p<0.001).

The study also reported a low incidence of agenesis, with bilateral agenesis of the FS recorded in 1.27% of cases and unilateral agenesis in 3.78% on the right side and 0.63% on the left side.

No significant age-related correlations were observed in the dimensions and volumes of the three paranasal air sinuses.

## Discussion

One of the foremost responsibilities of forensic anthropologists is to establish a biological profile of human remains, which involves assessing and identifying characteristics such as age, gender, ancestry, and stature. ^23^ As the population structure changes with the influx of immigrants from diverse geographic regions, relying on population-specific data from previous groups is no longer feasible. This shift in demographics has resulted in a more diverse population, and accurate identification of individuals through physical characteristics such as ancestry, age, and gender can be challenging. ^24, 25^ Thus, forensic anthropologists and other experts must stay up-to-date on the latest population trends and data to ensure that accurate identifications can be made, even for individuals from diverse backgrounds. ^21, 26^

According to several studies, accurate sex determination using the skull and pelvis is a well-established practice in forensic anthropology. It has been reported that skull both and pelvis can accurately determine sex especially for adults with rates of 95% and 90% respectively. ^27, 28^ This is because they are relatively imperishable owing to their challenging tissue components. ^29^

A study conducted by Arijit et al. ^30^ investigating the reliability of MS measurements obtained from CT scans and human skulls revealed that measurements of the maxillary sinus obtained from CT scans were similar to those obtained from human skulls. ^31^ The configuration of A study by Suman investigated FS among monozygotic twins. Their study showed that the configuration of the FS varies even among monozygotic twins, demonstrating its uniqueness and reliability for each individual. ^32^ Moreover, it has been shown that FS growth is completed by 18-20 years of age and seldom changes during life, except for trauma or pathology. ^33^ The current study showed no significant differences in FS dimensions and volume with age, possibly demonstrating that these dimensions had reached adult size.

**Figure 7.**
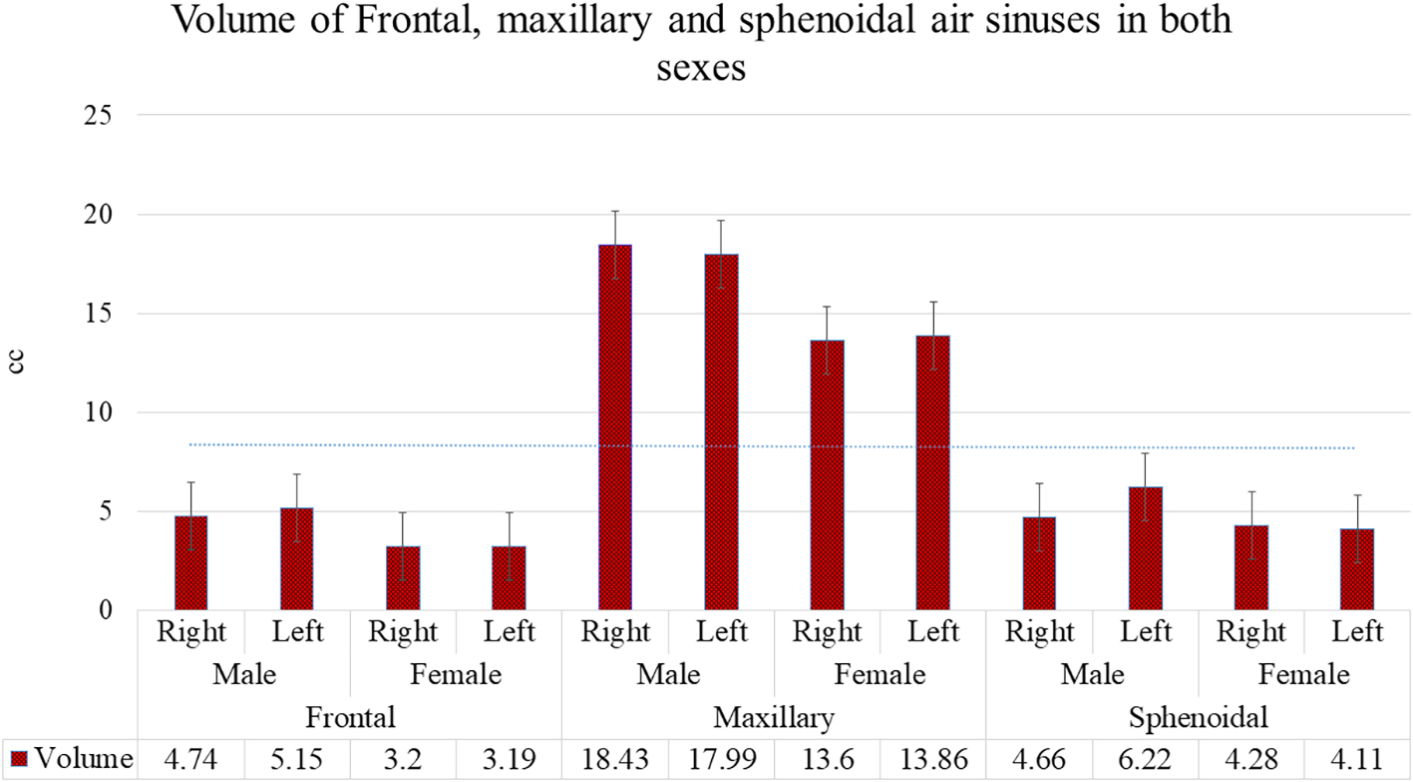
Comparison of dimensions of Frontal sinus between the previous and current study.

In the present study, the AP length, height, and volume of the FS showed mean values were greater in males than in females ( Figure 5 and Figure 6). Nutritional, hormonal, or muscular factors cause morphological differences between the sexes. ^34^ A CT scan study of the skulls of 53 Egyptian subjects by Motawei et al. ^35^ showed that the AP length, height, and width of the FS were more significant than those in our study ( Figure 7). Similarly, the height and width of the FS reported by Tatlisumak et al. ^14^ and the height, width, and volume reported by Ponde et al. ^36^ in a different population (Turkey and Brazil, respectively) showed relatively higher values in both sexes than in our study ( Figure 7). However, the FS volume in the dry skull studied by Ponde et al. ^36^ was significantly higher than that in our study, while Karkas et al. ^37^ and Fernandes et al. ^38^ reported FS volumes similar to our study. Various factors, such as population, genetics, and environment may play a role in these differences. ^24, 25, 39^ AP length, height, width, and volume of the MS showed that, in general, the size of the MS in our study was more extensive in males than in females ( Figure 5 and Figure 6). These findings were based on previous research reports wherein the size of the MS showed statistically significant values with a higher percentage of sexual dimorphism. ^40, 41^ However, compared to the Zulu and European populations, the height and width of the MS were larger in the present study in both sexes. ^25^ Previous CT scan studies on MS also suggest that maxillary sinus height is the best discriminant parameter for sexual dimorphism. ^42^ Among the modern Japanese population, Kawarai et al. reported that larger MS volumes correlated with a lower prevalence of sinusitis among their people. ^43^ No significant changes were observed in the dimensions and volumes of the MS correlated with age in our study. However, Schatz et al. observed that the volume of the maxillary and sphenoid sinuses increased for a period up to 15 years of age, after which it remained the same. ^44^

Likewise, the dimensions and volumes of the SS in our study did not show any significant correlation with age. However, all measurements and volumes of SS were larger in males ( Figure 5 and Figure 6) than in females, which is in agreement with the studies of Karakas & Kavakli and Spaeth et al. ^37, 45^ However, the AP length and width of male and female SS patients in the present study were significantly lower than those reported by Spaeth et al. ^45^

## Conclusion

The dimensions and volume of paranasal air sinuses can aid in estimating the sex of an individual in forensic anthropology. Furthermore, the data obtained in this study show the importance of population-specific measurements in sex determination.

CT scans can provide detailed and accurate information about anatomical features without distortion and with faster image processing time, making it a reliable technique for identifying unknown individuals, evaluating injuries, and determining the cause and manner of death. Thus, imaging is a reliable technique for identification in forensic anthropology, criminal investigations, and for documenting changes in population dynamics. In countries that do not allow autopsies for cultural or religious reasons, imaging can help evaluate possible injuries and clarify the manner and cause of death.

As technology continues to advance, it is probable that imaging will play an increasingly significant role in these fields, aiding in unraveling intricate scenarios, shedding light on complex cases, and providing answers regarding human history and identity.

## Ethics and consent

The study protocol was approved by the Manipal Academy of Higher Education through its Institutional Ethics Committee (reference number: 064 IEC 064/2010; on 09-03 2010), with participation being voluntary, anonymous, and risk-free.) in agreement with accepted international standards. Data collected were confidential and used exclusively for research purposes.

## Consent to participate

Written informed consent was obtained to publish the CT images of the participants.

## Authors’ contribution

**Conceptualization:** Soumya Vinod & Anu Vinod Ranade

**Data curation:** Soumya Vinod & Biswabina Ray

**Formal analysis:** Soumya Vinod & Anu Vinod Ranade

**Investigation:** Soumya Vinod

**Methodology:** Soumya Vinod, Anu Vinod Ranade, Rajalakshmi Rai

**Project administration:** Soumya Vinod

**Resources:** Soumya Vinod & Anu Vinod Ranade

**Supervision:** Biswabina Ray

**Software:** Soumya Vinod, Anu Vinod Ranade

**Validation:** Soumya Vinod, Anu Vinod Ranade, Biswabina Ray

**Visualization:** Rajalakshim Rai & Biswabina Ray

**Writing – original draft:** Soumya Vinod, Anu Vinod Ranade

**Writing – review & editing:** Soumya Vinod, Anu Vinod Ranade, Rajalakshmi Rai

## Data Availability

Zenodo: Dimensions and volumes of paranasal air sinuses, https://doi.org/10.5281/zenodo.13772363. ^46^ The project contains the following underlying data:
• File 1: 5a-results_Tables.doc
• File 2: measurements of maxillary sinus.xls
• File 3: measurements of sphenoidal sinus.xls
• File 4: measurements of the frontal sinus.xls
• File 5: STROBE_checklist_cross-sectional (1) - Copy.docx Data are available under the terms of the Creative Commons Attribution 4.0 International license (CC-BY 4.0).
