# Supplementary material for: Beyond the Surface: A Multidetector Computer Tomography Scan Investigation into Age and Gender Differences
Source: F1000Res. 2025 Jan 15;14:85. [Version 1] doi: 10.12688/f1000research.156445.1 (PMC11986412; doi:10.12688/f1000research.156445.1)
Supplement: Peer review history

# Reviewer report: Reviewer response for version 1

**DOI:** 10.5256/f1000research.171759.r367505

**Published:** 2025-04-28

**Authors:** Santosh Phajir Vishwanath Rai

**Affiliations:** 1 Department of Radiology, Kasturba Medical College, Mangalore, Manipal Academy of Higher Education, Manipal, Karnataka, India

**Overall Assessment:**

 This study presents an interesting evaluation of paranasal sinus measurements that can be applied in forensic science to estimate skeletal sex and age-at-death. The experiment provides valuable insights, but several key areas need to be addressed before the paper can be considered for accepting. While the strengths of the research are notable, certain minor revisions are necessary to enhance the manuscript's clarity and depth.

**Strengths:**

 The study focuses on paranasal sinus morphometry for sex determination, which is highly relevant in forensic anthropology, particularly in challenging cases. The use of CT scans enhances accuracy and non-invasiveness, allowing for precise measurements of complex anatomical structures. A relatively large sample size of 158 participants increases the statistical robustness and generalizability of the findings. The measurement of sinus volume adds a unique dimension often overlooked in similar research and reporting the incidence of agenesis provides valuable information for future studies and researchers.

**Points for Minor Revision:**

1. **Clarifying Novelty and Contribution:** Some sections require minor restructuring to improve overall flow. Revise these sections to ensure logical progression of ideas. While the study contributes to existing literature, a clearer statement outlining its unique contributions is needed. I suggest the authors to add a paragraph in the discussion to highlight novel aspects and how this finding is novel compared to previous studies.
2. **Adding Brief Anatomical Context:** Enhance clarity by including a brief overview of the paranasal sinus anatomy and a short section on their embryological development in the introduction.
3. **Discussing Limitations:** Include a section discussing the limitations of using CT scans, such as costs and equipment accessibility, and address potential pathologies that may affect sinus measurements.
4. **Expanding Comparisons and Clinical Implications:** The discussion would benefit from a deeper comparison with findings from other populations and an elaboration on clinical implications for forensic identification.
  1. **Improving Data Presentation:** Include a table in the results section displaying the range of measured dimensions and volumes for a comprehensive overview of the data.

Is the work clearly and accurately presented and does it cite the current literature?

Yes

If applicable, is the statistical analysis and its interpretation appropriate?

Yes

Are all the source data underlying the results available to ensure full reproducibility?

Yes

Is the study design appropriate and is the work technically sound?

Yes

Are the conclusions drawn adequately supported by the results?

Yes

Are sufficient details of methods and analysis provided to allow replication by others?

Yes

Reviewer Expertise:

General Radiology, Cross sectional Radiology

I confirm that I have read this submission and believe that I have an appropriate level of expertise to confirm that it is of an acceptable scientific standard, however I have significant reservations, as outlined above.

# Reviewer report: Reviewer response for version 1

**DOI:** 10.5256/f1000research.171759.r375304

**Published:** 2025-04-10

**Authors:** Nermine Nosseir

**Affiliations:** 1 Gulf Medical University, Ajman, United Arab Emirates

This study examines the use of multidetector computed tomography (CT) scans in assessing sex- and age-based differences in the dimensions and volumes of frontal, maxillary, and sphenoid sinuses. The study is a retrospective cross-sectional analysis of cranial CT scans from 158 adult patients (79 males, 79 females).  DICOM software was used by the  researchers for the following measurements; anteroposterior length, height, width, and volume of sinuses across axial, coronal, and sagittal planes. Statistical analysis was conducted by using Pearson’s correlation and  t-tests. The study showed that the sinus dimensions and volumes were significantly larger in males than females, but no significant correlation was observed with age. The findings in the study support the potential of CT-based morphometric analysis in sex determination for forensic purposes where traditional methods are limited especially in cases where fragmented remains are involved. The authors highlighted the importance of population-specific data and underscored CT imaging’s non-invasive advantage in forensic identification, particularly in regions where autopsies are restricted.

 The  methodology of this research is clearly structured and follows the  STROBE guidelines through utilizing a well-defined retrospective cross-sectional design. The sample size is balanced . High-resolution imaging with a 64-row detector CT scanner and isotropic data allowed precise, reproducible measurements across the planes. The use of DICOM software for morphometry and volumetric analysis enhances the objectivity. However, the reliance on retrospective clinical data may introduce bias of the sampling. In the present study the sinus dye injection provided volume estimation but details on validation or interobserver reliability for this step are lacking and we recommend that it should be clarified.

Is the work clearly and accurately presented and does it cite the current literature?

Yes

If applicable, is the statistical analysis and its interpretation appropriate?

Yes

Are all the source data underlying the results available to ensure full reproducibility?

Yes

Is the study design appropriate and is the work technically sound?

Yes

Are the conclusions drawn adequately supported by the results?

Yes

Are sufficient details of methods and analysis provided to allow replication by others?

Partly

Reviewer Expertise:

Gross and microscopic anatomy - Embryology- Congenital anomalies- Medical Education

I confirm that I have read this submission and believe that I have an appropriate level of expertise to confirm that it is of an acceptable scientific standard.

# Reviewer report: Reviewer response for version 1

**DOI:** 10.5256/f1000research.171759.r375298

**Published:** 2025-04-04

**Authors:** Vijay Paul Samuel

**Affiliations:** 1 Ras Al Khaimah Medical and Health Sciences University,, Ras Al Khaimah, United Arab Emirates

The manuscript is well written and addresses an important challenge faced by many forensic science specialist i.e to identification of people after a calamity. barring few minor spelling/grammatical errors the article can be accepted as it is. In the abstract the word non invasive has been wrongly hyphenated.

 It is better to stick to the term males uniformly rather than men which has been used at some instances. Kindly clarify if reformatted is same as reformation.

 Need to clarify how the investigators made sure that in the CT image that was available for measurement, the section had passed at maximum ap length , width and height.

Is the work clearly and accurately presented and does it cite the current literature?

Yes

If applicable, is the statistical analysis and its interpretation appropriate?

Yes

Are all the source data underlying the results available to ensure full reproducibility?

Yes

Is the study design appropriate and is the work technically sound?

Yes

Are the conclusions drawn adequately supported by the results?

Yes

Are sufficient details of methods and analysis provided to allow replication by others?

Yes

Reviewer Expertise:

Toxicity studies ,epidemiology,  anatomy and embryology.

I confirm that I have read this submission and believe that I have an appropriate level of expertise to confirm that it is of an acceptable scientific standard.

# Reviewer report: Reviewer response for version 1

**DOI:** 10.5256/f1000research.171759.r367506

**Published:** 2025-02-26

**Authors:** Maia Casna

**Affiliations:** 1 Leiden University, Leiden, The Netherlands

This study presents an interesting evaluation of whether paranasal sinus measurements can be applied in forensic science to accurately estimate skeletal sex and age-at-death. The experiment provides valuable insights, but several key areas need to be addressed before the paper can be considered for indexing.

**Major Points for Improvement:**

1. As presented in the discussion section, a substantial body of research has already explored the correlation between paranasal sinus size and skeletal sex. This is expected, given that the sinuses are part of the skull, which exhibits significant sexual dimorphism. It remains unclear how the authors' findings contribute new insights to the existing literature. I am not suggesting that these results are unimportant, but a clearer discussion is needed to highlight their novelty and significance.
2. The manuscript does not include a section explaining the anatomy and embryology of the paranasal sinuses, which is crucial for interpreting the results. A more detailed anatomical background would also strengthen the contextualization of findings. For example, the rationale behind expecting a correlation between sinus size and age needs further clarification, given that sinus development is largely complete by the time an individual reaches adult age. Without a clearer explanation, it is difficult to understand the relevance and utility of this analysis.

As a general reccomendation, the manuscript would benefit from careful proofreading, as there are multiple spelling errors and instances of repeated sentences. Please ensure that all inconsistencies are addressed before resubmission. In addition, the introduction and discussion sections should be restructured for clarity. Some topics (e.g., the presence of foreign bodies in the sinuses, human migration) are mentioned without a clear link to the main research question or analysis. These connections should be explicitly explained.

 Finally, I am sure that, with further refinement, this paper could make a valuable contribution to forensic literature on the paranasal sinuses. I strongly encourage the authors to revise and resubmit after addressing these critical points.

Is the work clearly and accurately presented and does it cite the current literature?

Partly

If applicable, is the statistical analysis and its interpretation appropriate?

Partly

Are all the source data underlying the results available to ensure full reproducibility?

Yes

Is the study design appropriate and is the work technically sound?

Yes

Are the conclusions drawn adequately supported by the results?

Partly

Are sufficient details of methods and analysis provided to allow replication by others?

No

Reviewer Expertise:

Bioarchaeology, anatomy and embryology of the paranasal sinuses. I suggest reaching out to a forensic professional for further comments on the contribution these data give to forensic science.

I confirm that I have read this submission and believe that I have an appropriate level of expertise to state that I do not consider it to be of an acceptable scientific standard, for reasons outlined above.
